# Anticoccidial Efficacy of Solid-Dispersion Formulations Containing *Curcuma longa* and *Piper longum* Extracts Against *Eimeria tenella* Infection in Broiler Chickens

**DOI:** 10.3390/vetsci13070675

**Published:** 2026-07-11

**Authors:** Nisachon Apinda, Wasana Chaisri, Terdsak Yano, Suwit Chotinan, Thanaporn Eiamsam-ang, Saruda Tiwananthagorn, Panuwat Yamsakul

**Affiliations:** Department of Veterinary Medicine, Faculty of Veterinary Medicine, Chiang Mai University, Chiang Mai 50200, Thailand; nisachon.a@cmu.ac.th (N.A.); wasana.ch@cmu.ac.th (W.C.); terdsak.yano@cmu.ac.th (T.Y.); suwit.c@cmu.ac.th (S.C.); thanaporn.e@cmu.ac.th (T.E.-a.); saruda.t@cmu.ac.th (S.T.)

**Keywords:** coccidiosis, *Eimeria tenella*, phytogenic extracts, solid dispersion, *Curcuma longa*, *Piper longum*, broiler chickens, anticoccidial efficacy

## Abstract

Coccidiosis is one of the most economically important parasitic diseases in poultry, causing intestinal damage, impaired nutrient utilization, and substantial production losses. The extensive use of anticoccidial drugs has raised concerns regarding drug resistance, chemical residues in poultry products, and the environmental burden associated with veterinary drug use, highlighting the need for complementary control strategies. This study evaluated the anticoccidial efficacy of phytogenic formulations containing *Curcuma longa* and *Piper longum* extracts prepared using a solid-dispersion technique designed to enhance the dissolution characteristics of poorly water-soluble phytogenic compounds. The in vitro activity of the formulations was evaluated against mixed *Eimeria* spp. field-isolated oocysts using scanning electron microscopy (SEM) to examine ultrastructural alterations, whereas in vivo efficacy was investigated in broiler chickens experimentally challenged with *Eimeria tenella*. Cecal lesion scores, oocyst shedding, and growth performance were evaluated as indicators of infection severity and treatment response. SEM observations revealed marked structural damage to mixed *Eimeria* spp. oocysts following exposure to the phytogenic formulations. In the animal trial, all phytogenic treatment groups exhibited lower cecal lesion scores and reduced oocyst shedding than the infected control group. Among the phytogenic formulations evaluated, the combination containing 6 g/kg *P. longum* (T3) tended to produce the lowest numerical lesion score and oocyst shedding, although differences among phytogenic treatments were limited. Although some treatment groups also exhibited numerically favorable growth performance under challenge conditions, these observations should be interpreted cautiously because the study was designed primarily as a preliminary evaluation of anticoccidial efficacy, and feed intake and feed conversion ratio were assessed without independent pen-level replication. The observed biological responses may be associated, in part, with the improved dissolution characteristics afforded by the solid-dispersion formulation; however, this proposed mechanism was not directly investigated in the present study. In conclusion, solid-dispersion formulations containing *C. longa* and *P. longum* extracts demonstrated promising anticoccidial activity against experimental *E. tenella* infection and support further investigation as promising phytogenic candidates for complementary coccidiosis control strategies in broiler chickens.

## Simple Summary

Coccidiosis is a common parasitic disease in poultry that damages the intestinal tract and causes substantial economic losses worldwide. Although anticoccidial drugs are widely used for disease control, concerns regarding drug resistance and chemical residues have increased interest in complementary control strategies. This study evaluated phytogenic formulations containing turmeric (*Curcuma longa*) and long pepper (*Piper longum*) extracts prepared using a solid-dispersion technique designed to improve the dissolution of bioactive compounds. The formulations caused structural damage to *Eimeria* oocysts, reduced intestinal lesion scores, and decreased oocyst shedding in broiler chickens experimentally infected with *Eimeria tenella* compared with the infected control group. Among the tested formulations, the combination containing 6 g/kg *P. longum* (T3) showed the most favorable numerical anticoccidial response. Although some treatments also exhibited numerically favorable growth performance under challenge conditions, these observations should be interpreted cautiously because this study was designed as a preliminary efficacy evaluation. Overall, the findings support further investigation of solid-dispersion phytogenic formulations as potential complementary approaches for coccidiosis control in broiler chickens.

## 1. Introduction

Coccidiosis is one of the most economically important parasitic diseases affecting the poultry industry worldwide, particularly in intensive broiler production systems. The disease is caused by protozoan parasites of the genus *Eimeria*, comprising at least nine species capable of infecting chickens. Among these, *Eimeria acervulina*, *E. brunetti*, *E. maxima*, *E. necatrix*, and *E. tenella* are considered the most pathogenic, causing diarrhea, dehydration, impaired growth, poor feed efficiency, and increased mortality [1,2,3,4]. Infection occurs following ingestion of sporulated oocysts, after which the parasites undergo intracellular development within intestinal epithelial cells, leading to extensive mucosal damage, impaired nutrient absorption, and reduced productive performance [5,6].

Coccidiosis continues to impose substantial economic losses on the poultry industry through decreased growth performance, increased feed conversion ratio, mortality, and treatment costs, with global losses estimated to exceed USD 2–3 billion annually [1,7]. Current control strategies rely primarily on anticoccidial drugs and vaccination [8,9,10]; however, the extensive use of chemotherapeutic agents has contributed to the emergence of drug-resistant Eimeria strains and increasing concerns regarding drug residues and food safety [11,12]. In addition, increasingly stringent regulations governing antimicrobial and anticoccidial drug use, together with growing consumer demand for residue-free poultry products, have intensified the search for sustainable complementary approaches to coccidiosis control [13,14].

Phytogenic feed additives have attracted increasing attention as potential alternatives or complementary approaches because many plant-derived compounds possess antimicrobial, antioxidant, anti-inflammatory, and immunomodulatory properties that may support intestinal health during enteric infections [15,16]. Among these, *Curcuma longa* and *Piper longum* are rich sources of curcuminoids and piperine, respectively, which have been widely investigated for their diverse biological activities. Previous studies have demonstrated beneficial effects of these plant extracts on gut health, intestinal microbiota, and productive performance in poultry [15,17,18]. However, evidence supporting their efficacy against protozoan infections, particularly avian coccidiosis, remains limited.

The principal bioactive compounds of *C. longa* and *P. longum* possess complementary biological properties that may be relevant to the pathogenesis of coccidiosis. Curcuminoids have been reported to exhibit antioxidant and anti-inflammatory activities that may contribute to maintaining intestinal epithelial integrity, whereas piperine has been reported to enhance the intestinal absorption of certain poorly water-soluble compounds [1,17]. These complementary characteristics provide a rationale for evaluating combined formulations of the two extracts; however, whether such combinations improve anticoccidial efficacy under experimental *Eimeria* infection has not been clearly established. The anticoccidial activity of phytogenic compounds has also been proposed to involve both direct and indirect mechanisms, including disruption of parasite development, modulation of inflammatory responses, reduction in oxidative stress, and preservation of intestinal function [19]. Nevertheless, many of these mechanisms remain hypothetical because they have not been directly demonstrated in vivo.

Despite their promising biological activities, practical application of many phytogenic compounds is limited by poor aqueous solubility and unfavorable dissolution characteristics, particularly for curcuminoids. Solid-dispersion technology has been widely employed to improve the dissolution behavior of poorly water-soluble compounds, including curcumin and other phytogenic substances [20,21]. In this approach, active compounds are molecularly dispersed within a hydrophilic carrier matrix, thereby improving their physicochemical characteristics. Among the available manufacturing techniques, hot-melt processing provides a simple solvent-free approach suitable for developing feed additive formulations.

Although numerous studies have investigated the antimicrobial, antioxidant, and growth-promoting properties of *C. longa* and *P. longum*, most have evaluated conventional herbal extracts without considering formulation strategies that may improve their physicochemical properties. Consequently, limited information is available regarding the application of solid-dispersion technology to enhance the anticoccidial efficacy of these phytogenic compounds in broiler chickens. Therefore, the novelty of the present study lies not only in evaluating the biological activity of *C. longa* and *P. longum* extracts but also in investigating whether formulation as a solid-dispersion system can enhance their biological performance during experimental coccidial infection.

Based on these considerations, we hypothesized that incorporating *C. longa* and *P. longum* extracts into a solid-dispersion formulation would improve the dissolution characteristics of the phytogenic compounds and thereby enhance their anticoccidial activity under experimental *Eimeria* infection. Therefore, the present study aimed to evaluate the anticoccidial efficacy of solid-dispersion formulations containing *C. longa* and *P. longum* extracts in broiler chickens experimentally challenged with *Eimeria tenella*. The study combined an in vitro evaluation using scanning electron microscopy to examine ultrastructural alterations in mixed *Eimeria* spp. field-isolated oocysts with an in vivo challenge model using *E. tenella*. Cecal lesion scores, oocyst shedding, histopathological changes, and growth performance were subsequently evaluated to determine the preliminary efficacy of these formulations as phytogenic candidates for complementary coccidiosis control in broiler chickens.

## 2. Materials and Methods

### 2.1. Preparation and Characterization of Herbal Extracts and Solid-Dispersion Formulations

The major bioactive compounds in the herbal extracts were quantified by high-performance liquid chromatography (HPLC) prior to formulation to ensure batch consistency and extract standardization. HPLC analysis was performed using an in-house analytical method developed from the analytical procedures described in the United States Pharmacopeia (USP) monographs for turmeric and piperine, with minor modifications to accommodate the available laboratory instrumentation. Separation was achieved on a reversed-phase C18 column. Curcuminoids were analyzed using an acetonitrile–aqueous acetic acid mobile phase with UV detection at 425 nm, whereas piperine was analyzed using a methanol-water mobile phase with UV detection at 340 nm. Curcumin, demethoxycurcumin, bis-demethoxycurcumin, and piperine were identified and quantified using authenticated reference standards. Calibration curves were prepared from serial standard solutions before sample analysis, and the concentrations of the major phytochemicals were expressed as percentage (*w*/*w*) of the crude extracts.

Phytochemical characterization confirmed curcumin as the principal constituent of Curcuma longa extract and piperine as the predominant alkaloid in Piper longum extract, consistent with previous reports [22,23,24].

Solid-dispersion formulations were prepared using the hot-melt method according to a previously established formulation protocol that had been optimized through preliminary formulation-development studies. Briefly, crude herbal extracts were mixed with polyvinylpyrrolidone K30 (PVP K30) at an extract-to-carrier ratio of 1:3 (*w*/*w*), which was selected based on preliminary evaluations of formulation stability, powder characteristics, and aqueous dispersibility [25,26]. The mixture was heated at 80 °C for approximately 15 min under continuous mixing until a homogeneous molten mass was obtained. These processing conditions are consistent with previously reported methods for preparing curcumin-based solid dispersions without significant thermal degradation [20,27]. The molten mass was allowed to cool to room temperature before being pulverized and sieved to obtain a homogeneous solid-dispersion powder.

**Figure 1:**
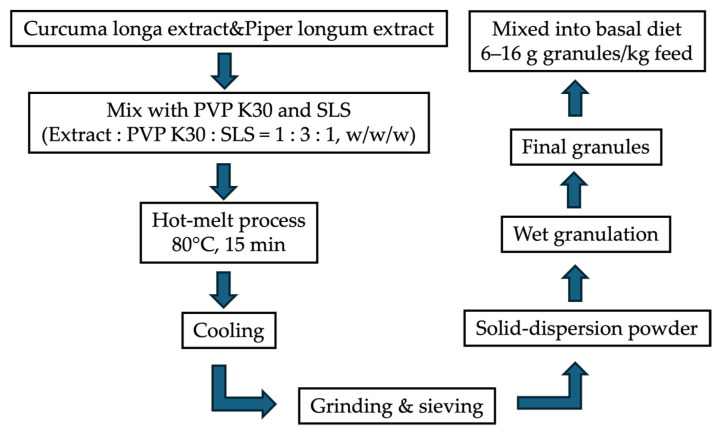
A schematic figure illustrating the preparation of the solid-dispersion granules and the relationship between the formulation components and the dietary inclusion levels has been added to improve the reproducibility and clarity of the experimental procedures.

The resulting powder was subsequently converted into feed-grade granules using a wet granulation process to improve flowability, handling characteristics, and uniform distribution in the experimental diets. Microcrystalline cellulose (Avicel PH101) and corn starch were used as excipients, whereas ethanol served only as a temporary granulating solvent. Following granulation, the products were dried under controlled conditions until a constant weight was achieved to facilitate complete evaporation of ethanol before biological evaluation. A schematic overview of the formulation procedure and dietary inclusion strategy is presented in Figure 1.

Unless otherwise specified, all formulation levels reported in this study (g/kg feed) refer to the final solid-dispersion granules incorporated into the experimental diets rather than crude herbal extracts or purified bioactive compounds. The final granules consisted of crude herbal extract, PVP K30, and sodium lauryl sulfate (SLS) at a ratio of 1:3:1 (*w*/*w*/*w*). This formulation was selected from a previously completed formulation-development study in which dissolution characteristics, physicochemical properties, chemical stability, and preliminary biological activity had been systematically evaluated before application in the present in vivo study.

### 2.2. In Vitro Anticoccidial Activity by Scanning Electron Microscopy (SEM)

For the in vitro assay, the solid-dispersion formulation consisting of crude herbal extract, polyvinylpyrrolidone K30 (PVP K30), and sodium lauryl sulfate (SLS) at a ratio of 1:3:1 (*w*/*w*/*w*) was suspended at a final concentration of 15 mg/mL. This concentration was selected based on our previous formulation-development study, in which it provided satisfactory dispersion characteristics and consistent visualization of oocyst ultrastructural alterations by scanning electron microscopy (SEM).

Oocysts used for SEM were collected from naturally infected chickens and therefore consisted of mixed *Eimeria* spp. field isolates. Consequently, the SEM assay was designed to qualitatively evaluate structural alterations of the oocyst wall following exposure to the phytogenic formulations rather than to determine species-specific susceptibility.

Fresh fecal samples containing unsporulated oocysts were collected and purified using standard procedures. The oocysts were sporulated in 2.5% (*w*/*v*) potassium dichromate (K_2_Cr_2_O_7_) under aerated conditions at room temperature for 48–72 h to obtain infective (sporulated) oocysts [21,28]. Before use, sporulated oocysts were washed repeatedly with sterile phosphate-buffered saline (PBS) to remove residual potassium dichromate.

Sporulated oocysts were incubated with the solid-dispersion formulations at 37 °C for 1 h, whereas oocysts incubated with sterile 0.9% normal saline solution (NSS) served as the negative control. Each treatment was prepared independently in triplicate.

Following incubation, samples were centrifuged at 1500 rpm for 5 min, washed with sterile PBS, and fixed in 2.5% (*v*/*v*) glutaraldehyde at 37 °C for 2 h. The fixed oocysts were subsequently dehydrated, dried using a critical-point dryer, sputter-coated with gold, and prepared for SEM examination according to a previously described protocol with slight modifications [29]. Samples were examined using a scanning electron microscope operated at an accelerating voltage of 15 kV with magnifications ranging from 2000× to 10,000×.

Morphological alterations were evaluated qualitatively based on changes in oocyst surface integrity, wall structure, deformation, and evidence of structural disruption, following previously published criteria for assessing antiparasitic activity [30,31]. These qualitative SEM observations were used solely to provide visual evidence supporting oocyst structural damage following treatment and were not subjected to inferential statistical analysis. To facilitate a standardized comparison of morphological damage among treatments, a separate semi-quantitative ordinal scoring system was additionally applied. A semi-quantitative scoring system was used to classify the degree of morphological damage as follows:

0 = normal morphology with intact wall;

1 = mild surface irregularities;

2 = moderate deformation or partial wall damage;

3 = severe structural disruption or rupture.

This scoring system was adapted from previously reported methods for evaluating ultrastructural damage in protozoan parasites and coccidian species using electron microscopy [30,32,33]. The resulting scores represent ordinal data describing the severity of morphological damage rather than direct quantitative measurements of parasite viability or anticoccidial efficacy. Accordingly, these ordinal scores were analyzed using appropriate non-parametric statistical methods as described in Section 2.9, whereas the accompanying SEM images were interpreted qualitatively as supportive morphological evidence only.

### 2.3. In Vivo Experimental Design

The in vivo experiment was conducted using a completely randomized design (CRD). A total of 74 one-day-old Cobb 500 broiler chicks of mixed sex were obtained from a commercial hatchery in Northern Thailand. Sample size was estimated using G*Power software (version 3.1.9.7) for one-way ANOVA with seven experimental groups. Based on a significance level (α) of 0.05 and statistical power of 0.80, approximately 10–11 birds per treatment were required. Accordingly, eleven birds were allocated to each phytogenic treatment group, whereas ten birds were assigned to each control group. Individual birds were considered the experimental units for body weight, lesion score, and oocyst shedding measurements. All experimental procedures were approved by the Ethics Committee of the Faculty of Veterinary Medicine, Chiang Mai University (Approval No. S01/2562; 20 February 2019).

This study was designed as a preliminary proof-of-concept evaluation of phytogenic anticoccidial formulations. Accordingly, treatment groups receiving *Curcuma longa* extract alone were not included. The phytogenic formulations and dietary inclusion levels were selected based on a previously completed formulation-development study [34], in which solid-dispersion formulations of *Curcuma longa* and *Piper longum* extracts were optimized with respect to physicochemical characteristics, handling properties, and preliminary biological performance. The selected inclusion levels also considered previous feeding observations and practical feed palatability.

Birds were randomly assigned to seven experimental groups:

NC (negative control): Uninfected and untreated (*n* = 10);

PC (positive control): Infected and treated with amprolium hydrochloride (*n* = 10);

IC (infected control): Infected and untreated (*n* = 10);

T1: Infected + combined formulation containing 8 g/kg Piper longum and 8 g/kg Curcuma longa (i = 11);

T2: Infected + combined formulation containing 8 g/kg Piper longum and 16 g/kg Curcuma longa (*n* = 11);

T3: Infected + Piper longum formulation at 6 g/kg (*n* = 11);

T4: Infected + Piper longum formulation at 8 g/kg (*n* = 11).

Unless otherwise specified, all dietary inclusion levels refer to the final solid-dispersion granules incorporated into the experimental diets rather than crude herbal extracts or purified active compounds.

Birds received a nutritionally balanced basal starter diet formulated to meet the nutrient requirements of broiler chickens from 0 to 21 days of age, and the ingredient composition and calculated nutrient contents are presented in Table 1. Nutrient composition was formulated to meet or exceed the recommendations of the National Research Council (NRC, 1994) [35]. The vitamin–mineral premix supplied per kilogram of diet is described in Table 1.

All birds were individually identified using wing tags throughout the experimental period. Individual body weight was recorded at the beginning and end of the experiment. Feed intake was measured daily on a pen basis by recording feed offered and feed remaining for each treatment group, after which average feed intake per bird was calculated. Mortality was monitored and recorded daily throughout the study.

### 2.4. Housing and Management

All birds were raised in an open-sided experimental poultry house at the experimental animal facility, Faculty of Veterinary Medicine, Chiang Mai University. Birds were housed in raised-floor cages (1.0 × 1.3 m), with stocking density maintained according to Thai agricultural standards (≤20 kg live weight/m^2^; each cage contained 10–11 birds). Environmental conditions were managed to approximate conventional broiler production, with an ambient temperature of 28 ± 2 °C, relative humidity of approximately 60 ± 5%, adequate natural ventilation, and a lighting program of 18 h light/day at an intensity of approximately 20 lux. Although the environmental conditions were standardized, the experiment was conducted in an experimental animal facility rather than under commercial farm conditions.

All birds received the experimentally formulated basal diet described in Table 1 throughout the study. Phytogenic formulations were incorporated directly into the feed, whereas the positive control group received amprolium hydrochloride via drinking water according to the manufacturer’s recommended dosage beginning at 5 days of age. This administration route reflects the practical use of amprolium under field conditions and differs from that of the phytogenic formulations, which were supplied continuously through the diet.

Standard biosecurity measures were implemented throughout the experiment, including restricted access, disinfectant footbaths, and routine sanitation procedures. Newcastle disease vaccination was administered by eye drop on days 7 and 17. The experimental period lasted 31 days. Individual body weight was recorded at day 0 and day 31, and body weight gain was calculated as the difference between initial and final body weights.

### 2.5. Eimeria Challenge

Sporulated oocysts of *Eimeria tenella* were obtained from the Faculty of Veterinary Medicine, Chulalongkorn University. Oocysts were maintained in 2% potassium dichromate solution prior to use. At 25 days of age, birds in all infected groups were orally challenged with 1 × 10^6^ sporulated oocysts per bird, a dose sufficient to induce clinical coccidiosis under experimental conditions [33,34].

### 2.6. Sample Collection and Oocyst Enumeration

At 6 days post-infection (31 days of age), birds were humanely euthanized by cervical dislocation. Cecal contents were collected and suspended in 2% (*w*/*v*) potassium dichromate solution for oocyst counting. Oocyst shedding was quantified using standard counting techniques and expressed as oocysts per gram (OPG) of feces.

### 2.7. Gross Lesion Scoring

Gross lesion scoring of the cecum was performed in all birds at 6 days post-infection to evaluate the severity of coccidial infection caused by *Eimeria tenella*. The scoring system was based on the standard method described by Johnson and Reid [36] and further detailed by Conway and McKenzie [37], with slight modifications.

Lesions were scored on a scale from 0 to 4 according to the severity of pathological changes observed in the cecum, as follows:

Score 0: No visible lesions; normal thin cecal wall with distinct longitudinal grooves and mucosal folds; homogeneous creamy content with no blood or solid debris.

Score 1: Mild petechial hemorrhages scattered on the cecal wall; normal cecal contents.

Score 2: Moderate hemorrhages with slight thickening of the cecal wall; presence of blood in the contents, with some normal content remaining.

Score 3: Severe hemorrhages with markedly thickened cecal wall; cecal contents appear as clotted masses mixed with blood.

Score 4: Severe distension of the cecum with caseous or blood-filled cores; absence of normal fecal material.

Lesion scoring was performed independently by two trained evaluators under blinded conditions to minimize observational bias. In cases of discrepancy, the average score was used for analysis. Lesion scores were expressed as mean values for each experimental group. Although lesion scoring represents ordinal data, mean values are commonly reported in poultry coccidiosis studies to facilitate comparison among treatments, and statistical analysis was performed using appropriate non-parametric methods.

### 2.8. Oocyst Enumeration

The number of *Eimeria* spp. oocysts in cecal contents was determined using the McMaster counting technique according to the procedures described in Poultry Coccidiosis: Diagnostic and Testing Procedures. Briefly, approximately 1 g of cecal content was accurately weighed and homogenized with saturated sodium chloride solution at a ratio of 1:15 (*w*/*v*) using a mechanical mixer to ensure uniform distribution of oocysts. The suspension was thoroughly mixed immediately before counting. Oocyst counts were expressed as the number of oocysts per gram (OPG) of fresh excreta.

An aliquot of the homogenized suspension was loaded into both chambers of a McMaster counting slide, and oocysts were counted under a light microscope at 100× magnification. Counts from the two chambers were averaged before calculation. The OPG value was calculated by multiplying the mean chamber count by a factor of 40, based on the dilution factor and chamber volume. The detection limit of the method was approximately 50 OPG.

### 2.9. Statistical Analysis

All statistical analyses were performed using R software (version 4.4.1). Continuous variables were initially assessed for normality and homogeneity of variance before statistical analysis. Variables satisfying the assumptions of parametric analysis were analyzed using one-way analysis of variance (ANOVA), followed by Tukey’s honestly significant difference (HSD) test for multiple comparisons when appropriate. Results are presented as mean ± standard error of the mean (SEM), and statistical significance was declared at *p* < 0.05.

Cecal lesion scores, which represent ordinal data, were analyzed using the non-parametric Kruskal–Wallis test followed by Dunn’s multiple comparison test with Holm adjustment for pairwise comparisons.

Growth performance variables measured at the individual bird level (body weight and body weight gain) were subjected to inferential statistical analysis. In contrast, feed intake and feed conversion ratio were determined at the pen level, with one pen assigned to each treatment. Because independent pen-level replication was not available, these variables are presented descriptively and were not subjected to inferential statistical analysis. Consequently, numerical differences in feed intake and feed conversion ratio should be regarded as descriptive observations and interpreted cautiously.

The SEM evaluation was designed as a qualitative morphological assessment. Individual oocysts and microscopic fields were considered observational units rather than independent biological replicates; therefore, SEM observations were used to provide supportive morphological evidence and were not subjected to inferential statistical analysis.

## 3. Results

### 3.1. Preparation and Characterization of Herbal Extracts

The phytochemical composition of the crude herbal extracts was determined by high-performance liquid chromatography (HPLC) prior to preparation of the solid-dispersion formulations to ensure batch consistency and extract standardization. Piperine was confirmed as the principal bioactive constituent of *Piper longum* extract, whereas curcumin, demethoxycurcumin, and bis-demethoxycurcumin were identified as the major curcuminoids present in *Curcuma longa* extract. The quantitative composition of these bioactive compounds is summarized in Table 2.

### 3.2. In Vitro Anticoccidial Activity (SEM)

**Figure 2:**
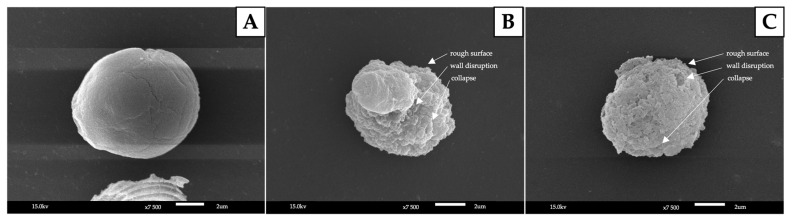
Scanning electron micrographs of *Eimeria* spp. oocysts: (**A**) control (untreated); (**B**) treated with *Piper longum* extract; (**C**) treated with *Curcuma longa* extract. Treated oocysts exhibited surface roughness, deformation, and structural damage compared to the smooth and intact surface observed in the control group.

The morphological changes in *Eimeria* spp. oocysts after exposure to herbal extracts were examined using scanning electron microscopy (SEM), as shown in Figure 2.

The oocysts in the control group (Figure 2A) exhibited a smooth and intact outer surface with a well-defined spherical structure. In contrast, oocysts treated with both *Piper longum* and *Curcuma longa* extracts (Figure 2B,C) showed notable structural alterations. The outer surface of the oocysts appeared roughened, irregular, and distorted, suggesting disruption of the oocyst wall integrity. Moreover, oocysts exposed to *Piper longum* extract (Figure 2B) demonstrated more severe surface damage compared to those treated with *Curcuma longa* extract (Figure 2C), as evidenced by increased surface collapse and irregularity. These observations indicate that the herbal extracts may exert direct anticoccidial effects by compromising the structural integrity of the oocyst wall.

A semi-quantitative scoring system was used to further evaluate the degree of morphological damage observed in oocysts (Table 2). Oocysts in the control group exhibited minimal structural alterations, with scores ranging between 0 and 1 (mean score = 0.32 ± 0.09). In contrast, oocysts treated with *Piper longum* and *Curcuma longa* extracts showed significantly higher damage scores, ranging from 2 to 3, with mean values of 2.56 ± 0.21 and 2.47 ± 0.18, respectively (*p* < 0.05). These results are consistent with the SEM observations, confirming that both herbal extracts induced substantial structural disruption of the oocyst wall.

### 3.3. In Vivo Effects of Treatments on Growth Performance in Broiler Chickens

Growth performance results are presented in Table 3. No significant differences in initial body weight were observed among groups (*p* > 0.05), indicating uniformity at the start of the experiment. At the end of the trial, significant differences were observed in final body weight, body weight gain, and average daily gain (ADG) (*p* < 0.05). The negative control (NC) group exhibited the highest growth performance overall, whereas the infected control (IC) group tended to show lower growth performance values than the NC group. Among the treatment groups, T3 demonstrated growth performance comparable to the positive control (PC), while T4 exhibited relatively lower performance among the treated groups (Table 4).

Feed intake and feed conversion ratio (FCR) are summarized in Table 5. Because feed intake was measured at the pen level with one pen assigned to each treatment, independent pen-level replication was not available. Consequently, feed intake and FCR are presented descriptively only, and no inferential statistical analysis was performed. The numerical values are provided to describe the observed responses under the experimental conditions and should not be interpreted as evidence of treatment effects.

### 3.4. Gross Lesion and Cecal Lesion Scores

**Figure 3:**
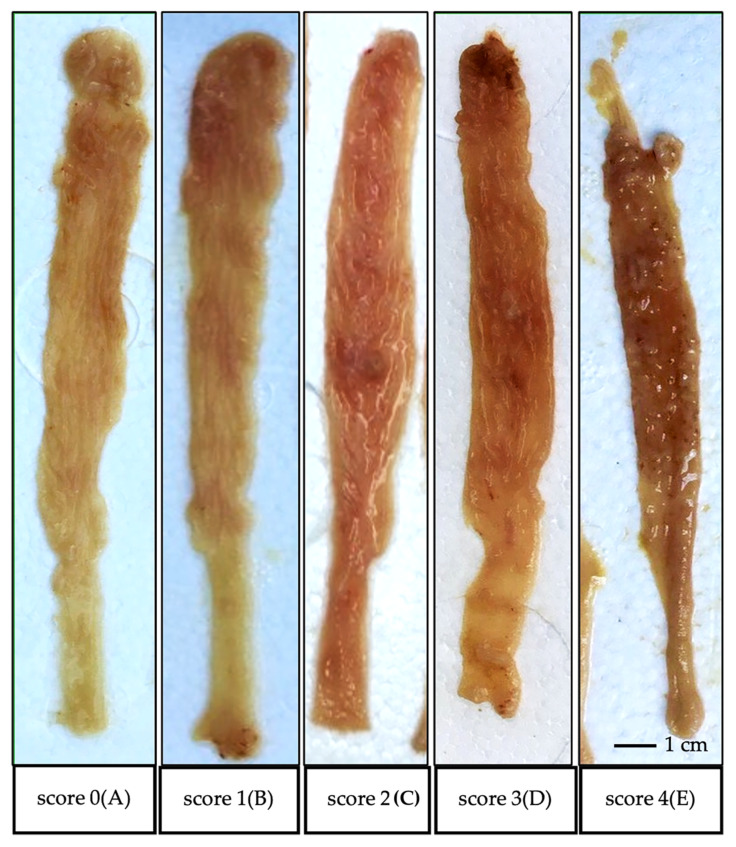
Representative gross lesion scoring of the cecum in broiler chickens infected with *Eimeria tenella*. Lesions were classified according to a modified scoring system based on Johnson and Reid [36] and Conway and McKenzie [37]: (**A**) score 0, normal cecum with no visible lesions; (**B**) score 1, mild petechial hemorrhages; (**C**) score 2, moderate hemorrhage and slight wall thickening; (**D**) score 3, severe hemorrhage with marked thickening and blood clots; (**E**) score 4, severe distension with caseous or blood-filled cores and absence of normal fecal content.

Representative gross lesions of the cecum are shown in Figure 3. Birds in the infected control (IC) group exhibited severe pathological changes, including hemorrhage, thickening of the cecal wall, and accumulation of blood-tinged contents. In contrast, the negative control (NC) group showed no visible lesions.

**Figure 4:**
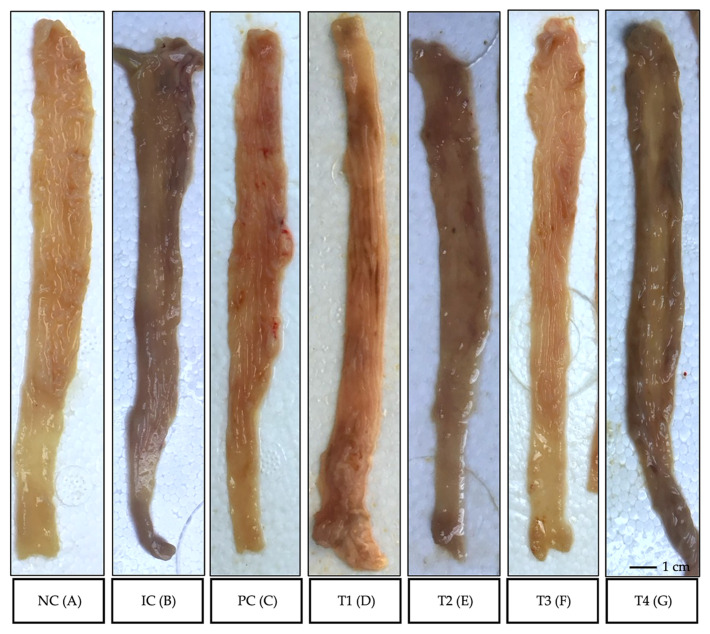
Representative gross morphology of the cecum in broiler chickens from different experimental groups at 6 days post-infection with *Eimeria tenella*. (**A**) Negative control (NC), showing normal cecal structure with no visible lesions; (**B**) Infected control (IC), exhibiting severe hemorrhage, thickening of the cecal wall, and abnormal contents; (**C**) Positive control (PC), showing mild lesions with partial recovery; (**D**–**G**) Treatment groups (T1–T4), demonstrating varying degrees of lesion severity, with T3 showing relatively mild pathological changes compared to other treatment groups.

The effects of treatments on lesion severity are summarized in Table 6 and Figure 4. Significant differences in lesion scores were observed among groups (*p* < 0.05). The IC group showed the highest lesion score (2.45 ± 0.53), indicating severe infection. Treatment groups exhibited reduced lesion severity compared to the IC group. Notably, the positive control (PC) and T3 groups showed significantly lower lesion scores (0.73 ± 0.11 and 0.68 ± 0.42, respectively), suggesting effective mitigation of intestinal damage. In contrast, T1 and T4 showed relatively higher lesion scores, indicating less pronounced protective effects.

### 3.5. Oocyst Shedding (Oocysts per Gram; OPG)

The effects of dietary treatments on oocyst shedding are presented in Table 7. No oocysts were detected in the negative control (NC) group throughout the experimental period. In contrast, the infected control (IC) group exhibited the highest oocyst shedding (5.74 × 10^6^ OPG), which was significantly greater than that of all treated groups (*p* < 0.05).

Administration of amprolium (PC) markedly reduced oocyst shedding compared with the infected control, confirming the effectiveness of the reference anticoccidial treatment. All phytogenic treatment groups also exhibited lower oocyst counts than the infected control. Among the phytogenic formulations, T3 (6 g/kg *Piper longum*) showed the lowest numerical oocyst shedding (1.39 × 10^6^ OPG), followed by T2 and T4, although differences among the phytogenic treatment groups were not statistically significant (*p* > 0.05). T1 showed a numerically higher oocyst count than the other phytogenic treatments.

Overall, all phytogenic formulations significantly reduced oocyst shedding compared with the infected control (*p* < 0.05), demonstrating measurable anticoccidial activity under the experimental conditions.

## 4. Discussion

Coccidiosis remains one of the most economically important parasitic diseases affecting the poultry industry because of its detrimental effects on intestinal integrity, nutrient utilization, growth performance, and the increasing concerns associated with anticoccidial drug resistance and chemical residues in poultry products [2,7,11,14]. Consequently, considerable attention has been directed toward phytogenic compounds as potential complementary approaches for coccidiosis control because of their reported antimicrobial, antioxidant, and anti-inflammatory properties [1,12,19]. In the present study, the formulated phytogenic preparations exhibited biological activity in both the in vitro and in vivo experiments. Exposure of mixed *Eimeria* spp. field-isolated oocysts to the phytogenic formulations resulted in marked ultrastructural alterations of the oocyst surface as observed by scanning electron microscopy (SEM), whereas broiler chickens experimentally challenged with *Eimeria tenella* exhibited lower cecal lesion scores and reduced oocyst shedding following phytogenic supplementation compared with the infected untreated control. Collectively, these findings provide complementary evidence supporting the anticoccidial potential of the formulated herbal extracts under the experimental conditions of the present study.

The present work differs from most previous studies in that the herbal extracts were incorporated into a solid-dispersion delivery system before biological evaluation. Poor aqueous solubility is one of the principal factors limiting the biological application of many phytogenic compounds, particularly curcuminoids, whose low dissolution rate and rapid metabolism substantially restrict oral availability [22,23,24]. Although piperine has frequently been used as a bio-enhancer, its own physicochemical characteristics may also limit dispersion in aqueous environments. These limitations have encouraged the development of formulation strategies designed to improve the dissolution behavior of poorly water-soluble phytogenic compounds.

Among the available formulation approaches, solid-dispersion technology has been widely recognized as an effective strategy for improving the dissolution characteristics of hydrophobic compounds by dispersing the active ingredients within a hydrophilic carrier matrix [26,27]. This process generally increases wettability, reduces crystallinity, and enhances the apparent surface area available for dissolution, thereby facilitating release of the active compounds in the gastrointestinal tract [26,27]. Previous pharmaceutical studies have demonstrated that solid-dispersion formulations can substantially improve the dissolution behavior and oral performance of curcumin compared with conventional preparations [27,29]. In addition, piperine has been reported to enhance intestinal absorption through modulation of membrane permeability and inhibition of drug-metabolizing enzymes, potentially increasing the biological availability of co-administered compounds such as curcumin [38,39].

Although dissolution behavior, physicochemical characteristics, and formulation stability had been optimized during our previous formulation-development study before conducting the present biological experiment, these parameters were not directly evaluated in the current investigation. Therefore, the improved biological responses observed in the treated groups may be associated, at least in part, with the enhanced dissolution characteristics provided by the solid-dispersion formulation; however, improvements in intestinal absorption, pharmacokinetics, or bioavailability were not directly measured in this study and should therefore be regarded as plausible rather than confirmed mechanisms.

The observed reductions in cecal lesion scores and oocyst shedding are consistent with previous reports describing the anticoccidial potential of phytogenic compounds [1,12,30]. Oocyst shedding represents one of the most informative indicators of parasite replication and environmental contamination, whereas lesion scores reflect the severity of intestinal damage induced during the parasite life cycle. In the present study, all phytogenic treatments reduced oocyst output relative to the infected control group, while lesion severity was also alleviated. Among the evaluated formulations, the treatment containing 6 g/kg *P. longum* (T3) produced the lowest numerical lesion score and oocyst shedding, although differences among the phytogenic formulations were generally limited. These findings suggest that the evaluated formulations were capable of suppressing parasite-associated intestinal pathology under the experimental challenge conditions.

The SEM observations provide supportive qualitative evidence for these in vivo findings. Structural alterations including deformation, surface disruption, and loss of normal oocyst wall integrity were consistently observed following exposure of mixed *Eimeria* spp. field-isolated oocysts to the phytogenic formulations. Similar ultrastructural damage has previously been associated with impaired sporulation, reduced infectivity, and decreased parasite viability in coccidian parasites [30,32,33]. Nevertheless, the SEM analysis performed in the present study was designed to provide qualitative morphological evidence rather than quantitative measurements of anticoccidial efficacy. Furthermore, because mixed field isolates of *Eimeria* spp. were used in the in vitro assay, species-specific susceptibility could not be determined. Consequently, conclusions regarding efficacy against *E. tenella* are based primarily on the results obtained from the controlled in vivo challenge experiment.

Although improvements in growth performance have frequently been reported following dietary supplementation with phytogenic feed additives, interpretation of growth responses in the present study should be undertaken with caution. Individual body weight measurements demonstrated that several treated groups maintained growth comparable to or numerically greater than that of the infected control group. However, feed intake and feed conversion ratio were measured at the pen level, with only one pen assigned to each treatment, precluding valid inferential statistical comparisons for these parameters. Consequently, the numerical differences observed in feed intake and feed conversion ratio should be regarded as exploratory observations rather than definitive evidence of treatment efficacy. This conservative interpretation is consistent with the preliminary design of the present study and avoids overinterpretation of production responses beyond the statistical strength of the experimental design.

The relatively low final body weights observed across all experimental groups also warrant consideration when interpreting the biological responses. Compared with commercial production standards for Cobb 500 broilers, birds in the present study exhibited lower growth performance at 31 days of age. Several factors likely contributed to this outcome, including the controlled experimental housing conditions, the deliberate *E. tenella* challenge, and the use of an experimental diet formulated specifically for research purposes rather than a commercial high-performance broiler diet. Collectively, these conditions were intended to establish a reproducible disease model rather than maximize productive performance. Therefore, the growth data should primarily be interpreted within the context of experimental infection rather than commercial production efficiency.

An interesting observation in the present study was that the combined phytogenic formulations did not consistently outperform the *Piper longum*-only treatments across all evaluated outcomes. Although the rationale for combining *Curcuma longa* and *Piper longum* was based on the complementary biological activities of curcuminoids and piperine together with the reported bio-enhancing effect of piperine [15,17,38], the treatment containing 6 g/kg *P. longum* (T3) produced the lowest numerical lesion scores and oocyst shedding. These findings suggest that increasing the proportion of *Curcuma longa* within the evaluated formulations did not necessarily result in proportionally greater biological responses under the present experimental conditions.

Several explanations may account for this observation. First, the biological activity of phytogenic compounds is not always dose-dependent, particularly when multiple bioactive constituents are combined. Interactions among phytochemicals may influence their physicochemical behavior, intestinal release characteristics, or local biological activity, resulting in responses that differ from those predicted based solely on individual components. Second, although piperine has been widely reported to enhance intestinal absorption of curcuminoids [38], the optimal ratio between curcumin and piperine within solid-dispersion formulations remains uncertain. Finally, the present study was not designed as a factorial experiment capable of independently evaluating the individual and interactive effects of the two herbal extracts. Therefore, the current data do not permit conclusions regarding additive or synergistic interactions between curcuminoids and piperine. Clarification of these relationships will require future studies incorporating individual *Curcuma longa* treatment groups together with appropriately replicated factorial experimental designs.

Previous investigations evaluating dietary supplementation with turmeric or long pepper have reported improvements in intestinal health, antioxidant capacity, immune responses, and productive performance in poultry [12,15,17]. Likewise, several studies have demonstrated reductions in lesion severity and oocyst shedding following administration of phytogenic compounds to *Eimeria*-infected chickens [1,12]. Nevertheless, most previous studies have evaluated conventional herbal powders or crude extracts without addressing the limitations associated with poor aqueous solubility of many phytogenic constituents. The present study extends this body of knowledge by evaluating standardized phytogenic extracts incorporated into a solid-dispersion delivery system before in vivo application. Consequently, the novelty of this work resides not only in demonstrating the biological activity of *Curcuma longa* and *Piper longum* extracts but also in exploring formulation technology as an approach for improving the practical application of phytogenic feed additives in poultry.

Despite these encouraging findings, the precise mechanisms responsible for the observed anticoccidial responses remain incompletely understood. Previous studies have suggested that curcuminoids may alleviate oxidative stress, modulate inflammatory responses, preserve epithelial integrity, and support intestinal health during coccidial infection, whereas piperine has been associated with improved intestinal absorption and enhanced biological availability of co-administered compounds [15,17,30]. However, the present study did not directly evaluate oxidative stress markers, inflammatory mediators, immune responses, intestinal morphology, or pharmacokinetic parameters. Consequently, these mechanisms should be considered biologically plausible explanations supported by previous literature rather than experimentally confirmed pathways in the present investigation. Future studies incorporating quantitative image analysis, including cecal wall morphometry and digital lesion assessment, would provide additional objective evidence supporting the gross pathological findings [40,41].

The findings of the present study should be interpreted in light of several limitations. First, this investigation was designed as a preliminary proof-of-concept study with a relatively small number of birds, and the experimental design did not include independent pen-level replication. Consequently, although individual bird measurements such as body weight, lesion scores, and oocyst shedding could be analyzed statistically, feed intake and feed conversion ratio were necessarily presented descriptively because these variables were measured at the pen level. Second, the experimental treatments did not include a *Curcuma longa*-only group. Consequently, although the combined formulations and *Piper longum*-based treatments demonstrated measurable biological activity, the independent contribution of curcuminoids could not be determined, nor could additive or synergistic interactions between the two herbal extracts be rigorously evaluated. Third, the present study primarily focused on biological efficacy and did not include direct measurements of dissolution behavior, intestinal absorption, pharmacokinetics, oxidative stress, inflammatory responses, immune modulation, or intestinal morphology. Therefore, mechanistic interpretations regarding the observed anticoccidial responses remain speculative and should be considered within the context of previous literature rather than direct experimental evidence.

An additional consideration is that the in vitro and in vivo experiments addressed different aspects of the biological response. The SEM assay employed mixed field isolates of *Eimeria* spp. to examine qualitative ultrastructural alterations of oocysts following exposure to the phytogenic formulations, whereas the animal experiment utilized a controlled *Eimeria tenella* challenge to evaluate anticoccidial efficacy under standardized conditions. Accordingly, the SEM observations should be regarded as supportive qualitative evidence demonstrating direct structural damage to *Eimeria* oocysts rather than species-specific confirmation of activity against *E. tenella.* The principal evidence supporting efficacy against *E. tenella* is derived from the reductions in lesion scores and oocyst shedding observed in the experimentally challenged broiler chickens [42,43].

Despite these limitations, the study possesses several important strengths. The phytogenic extracts were chemically standardized prior to formulation, thereby improving batch consistency throughout the biological experiments. Furthermore, the incorporation of formulation technology into the evaluation of phytogenic feed additives represents an important advancement beyond conventional studies employing crude herbal extracts alone. By combining phytochemical standardization, solid-dispersion formulation, qualitative ultrastructural evaluation, and controlled in vivo challenge, the present study provides an integrated assessment of the anticoccidial potential of Curcuma longa and Piper longum formulations. Although additional work is required to establish the underlying mechanisms and optimize formulation characteristics, the current findings provide an experimental foundation for subsequent translational studies.

Future investigations should therefore focus on validating these observations using larger, appropriately replicated experiments conducted under commercial production conditions. Inclusion of independent *Curcuma longa* treatment groups together with factorial experimental designs would enable rigorous evaluation of the individual contributions of curcuminoids and piperine as well as potential interactions between these phytogenic compounds. In addition, direct characterization of dissolution behavior, pharmacokinetic profiles, intestinal bioavailability, oxidative stress biomarkers, inflammatory mediators, immune responses, and intestinal histomorphology would substantially improve understanding of the mechanisms responsible for the observed biological effects. Such studies will be essential for determining whether solid-dispersion phytogenic formulations can be translated into practical feed additive strategies for sustainable coccidiosis control in commercial poultry production.

Collectively, the present findings provide a foundation for further investigation of formulation-based phytogenic strategies for coccidiosis control. Future studies should focus on validating these observations under commercial production conditions using adequately replicated experimental designs while incorporating mechanistic evaluations, pharmacokinetic characterization, and dose optimization studies. Such investigations will be essential for determining whether solid-dispersion phytogenic formulations can be translated into practical feed additives for sustainable coccidiosis management in poultry production.

## 5. Conclusions

This study demonstrated that solid-dispersion formulations containing *Curcuma longa* and *Piper longum* extracts exhibited measurable anticoccidial activity against experimental *Eimeria tenella* infection in broiler chickens. Compared with the infected untreated control, phytogenic supplementation reduced cecal lesion severity and oocyst shedding, while scanning electron microscopy provided supportive qualitative evidence of structural damage in mixed *Eimeria* spp. oocysts following exposure to the formulations. Together, these findings indicate that formulation of phytogenic extracts using a solid-dispersion approach represents a feasible strategy for enhancing their evaluation as anticoccidial feed additives.

The present study was designed as a preliminary proof-of-concept investigation; therefore, the findings should be interpreted within the context of its experimental design. Although some treatment groups exhibited numerically favorable growth performance under challenge conditions, these observations remain exploratory because feed intake and feed conversion ratio were determined without independent pen-level replication. Likewise, the biological mechanisms underlying the observed anticoccidial responses were not directly investigated, and the contribution of improved dissolution characteristics provided by the solid-dispersion formulation remains a plausible, but unconfirmed, explanation.

Overall, the present findings support further investigation of solid-dispersion phytogenic formulations as potential complementary approaches for coccidiosis control rather than validated alternatives to conventional anticoccidial drugs. Future studies should incorporate larger populations, appropriately replicated pen-level experimental designs, optimized dosage regimens, commercial production conditions, and mechanistic evaluations. In addition, inclusion of individual *Curcuma longa* and *Piper longum* treatment groups together with factorial experimental designs will be important for clarifying the independent contributions of each extract and determining whether additive or synergistic interactions contribute to the observed anticoccidial activity.

## Figures and Tables

**Table 1:** Ingredient composition and calculated nutrient content of the basal diet used during the starter period (0–21 days) (air-dried basis).

**Ingredients**	**Inclusion Level (%)**
Ground corn	55.00
Soybean meal (44% CP)	32.00
Fish meal	8.00
Vegetable oil/animal fat	3.00
Limestone, salt, vitamin–mineral premix	2.00
Total	100.00
**Nutrient Composition**	**Nutrient Composition**
Metabolizable energy (ME, kcal/kg)	3000
Crude protein (%)	22.00
Crude fat (%)	5.00
Crude fiber (%)	2.50
Calcium (%)	1.00
Available phosphorus (%)	0.42
Sodium (%)	0.16
Lysine (%)	1.20

Values are expressed on an as-fed basis. The basal diet was formulated specifically for this experiment to meet or exceed the nutrient requirements of broiler chickens recommended by the National Research Council (NRC, 1994) [35]. The same basal diet was provided to all experimental groups throughout the study, with phytogenic formulations incorporated into the diets according to the assigned treatments. Dietary inclusion levels refer to the final solid-dispersion granules rather than crude herbal extracts or purified bioactive compounds. Abbreviations: CP, crude protein; ME, metabolizable energy. Vitamin–mineral premix supplied per kilogram of diet: vitamin A, 12,000 IU; vitamin D_3_, 2500 IU; vitamin E, 30 IU; vitamin K_3_, 3 mg; vitamin B_1_, 2 mg; vitamin B_2_, 6 mg; vitamin B_6_, 4 mg; vitamin B_12_, 0.02 mg; niacin, 40 mg; pantothenic acid, 10 mg; folic acid, 1 mg; biotin, 0.15 mg; choline chloride, 500 mg; Fe, 80 mg; Cu, 8 mg; Mn, 80 mg; Zn, 60 mg; I, 0.35 mg; Se, 0.15 mg.

**Table 2:** Chemical characterization of the herbal extracts used in this study.

Herbal Extract	Major Active Compound	Content (% *w*/*w* of Crude Extract)	Analytical Method
*Piper longum*	Piperine	18.06 ± 0.40	HPLC
*Curcuma longa*	Curcumin	10.48 ± 0.55	
	Demethoxycurcumin	4.35 ± 0.20	
	Bis-demethoxycurcumin	4.50 ± 0.18	

Values are presented as mean ± SD (n = 3). The concentrations of the major bioactive compounds were determined by high-performance liquid chromatography (HPLC) prior to formulation and are expressed as percentage (*w*/*w*) of the crude extracts.

**Table 3:** Semi-quantitative scoring of morphological damage in *Eimeria* spp.

Treatment Group	Damage Score (Mean ± SD)
Control	0.32 ± 0.09 ^a^
*Piper longum* extract	2.56 ± 0.21 ^b^
*Curcuma longa* extract	2.47 ± 0.18 ^b^

Values are expressed as mean ± SD (n = 3) based on triplicate analytical determinations. Different superscript letters (a, b) within the same row indicate significant differences (*p* < 0.05). Note: Values represent mean ± SD from three independent fields, with 20 oocysts evaluated per field. SEM micrographs are presented as qualitative supportive evidence only. Statistical analysis was performed only for the semi-quantitative morphological damage scores.

**Table 4:** Effects of different treatments on growth performance parameters of broiler chickens experimentally challenged with *Eimeria tenella*.

Parameter	NC	IC	PC	T1	T2	T3	T4	*p*-Value
Initial weight (g)	42.00 ± 1.32	41.33 ± 1.85	43.40 ± 1.05	43.72 ± 0.55	43.81 ± 0.73	42.27 ± 0.72	42.72 ± 1.16	0.648
Final weight (g)	1093.57 ± 106.99 ^a^	897.33 ± 22.25 ^bc^	992.55 ± 55.82 ^ab^	903.63 ± 27.71 ^bc^	889.54 ± 62.90 ^bc^	1008.55 ± 41.05 ^ab^	836.81 ± 41.97 ^c^	0.048
Body weight gain (g)	1054.57 ± 107.45 ^a^	856.00 ± 23.81 ^bc^	949.10 ± 55.94 ^ab^	859.91 ± 27.65 ^bc^	845.72 ± 62.75 ^bc^	966.11 ± 40.67 ^ab^	794.09 ± 41.43 ^c^	0.046
ADG (g/day)	33.92 ± 3.46 ^a^	27.61 ± 0.76 ^bc^	30.61 ± 1.80 ^ab^	27.73 ± 0.89 ^bc^	27.28 ± 2.02 ^bc^	31.16 ± 1.31 ^ab^	25.61 ± 1.22 ^c^	0.046

Values are presented as mean ± SEM. Means within the same row with different superscript letters (a–c) differ significantly (*p* < 0.05). Growth performance was evaluated during the 10-day period following experimental *Eimeria tenella* challenge (days 21–31). Individual birds were considered the experimental units for body weight, body weight gain, and average daily gain (ADG). NC = non-infected, non-treated control; IC = infected, untreated control; PC = infected and treated with amprolium hydrochloride; T1 = solid-dispersion formulation containing 8 g/kg *Piper longum* and 8 g/kg *Curcuma longa*; T2 = solid-dispersion formulation containing 8 g/kg *Piper longum* and 16 g/kg *Curcuma longa*; T3 = solid-dispersion formulation containing 6 g/kg *Piper longum*; T4 = solid-dispersion formulation containing 8 g/kg *Piper longum*. Unless otherwise specified, dietary inclusion levels refer to the final solid-dispersion granules incorporated into the experimental diets.

**Table 5:** Feed intake and feed conversion ratio (FCR) of broiler chickens under different treatments. Values are presented descriptively because feed intake was measured at the pen level with one pen per treatment; therefore, no inferential statistical analysis was performed.

Parameter	NC	IC	PC	T1	T2	T3	T4
Total FI (g)	1924.11	2196.75	2337.78	2305.55	2317.90	2339.88	2304.82
ADFI (g/day)	62.07	70.86	75.41	74.37	74.77	75.48	74.35
FCR	1.83	2.57	2.46	2.68	2.74	2.42	2.90

NC = non-infected, non-treated control; IC = infected, untreated control; PC = infected and treated with amprolium hydrochloride; T1 = solid-dispersion formulation containing 8 g/kg *Piper longum* and 8 g/kg *Curcuma longa*; T2 = solid-dispersion formulation containing 8 g/kg *Piper longum* and 16 g/kg *Curcuma longa*; T3 = solid-dispersion formulation containing 6 g/kg *Piper longum*; T4 = solid-dispersion formulation containing 8 g/kg *Piper longum*. Unless otherwise specified, dietary inclusion levels refer to the final solid-dispersion granules incorporated into the experimental diets. Values represent single pen observations for each treatment; therefore, measures of variation (SEM) and inferential statistical analyses were not applicable.

**Table 6:** Effects of dietary treatments on cecal lesion scores in broiler chickens experimentally infected with *Eimeria tenella* at 6 days post-infection.

Parameter	NC	IC	PC	T1	T2	T3	T4	*p*-Value
cecal lesion scores	0.0 ± 0.0	2.45 ± 0.53 ^a^	0.73 ± 0.11 ^b^	1.52 ± 0.57 ^a^	1.38 ± 0.83 ^ab^	0.68 ± 0.42 ^b^	1.97 ± 0.98 ^a^	0.013

Values are presented as mean ± SEM. Means within the same row with different superscript letters (a, b) differ significantly (*p* < 0.05).

**Table 7:** Effects of dietary treatments on oocyst shedding (oocysts per gram; OPG) in broiler chickens experimentally infected with *Eimeria tenella*.

Treatment Group	OPG (×10^6^ Oocysts/g, Mean ± SEM)
NC	0.00 ± 0.00 ^a^
IC	5.74 ± 2.22 ^b^
PC	1.01 ± 0.69 ^c^
T1	3.21 ± 0.06 ^d^
T2	2.36 ± 0.49 ^cd^
T3	1.39 ± 0.16 ^d^
T4	2.90 ± 0.48 ^cd^
*p*-value	<0.001

Values are presented as mean ± SEM. Means with different superscript letters (a–d) within the same column differ significantly (*p* < 0.05).

## Data Availability

The original contributions presented in this study are included in the article. Further inquiries can be directed to the corresponding author.
